# Technology-Based Dietary Assessment in Youth with and Without Developmental Disabilities

**DOI:** 10.3390/nu10101482

**Published:** 2018-10-11

**Authors:** Michele Polfuss, Andrea Moosreiner, Carol J. Boushey, Edward J. Delp, Fengqing Zhu

**Affiliations:** 1 University of Wisconsin-Milwaukee, College of Nursing, Milwaukee, WI 53211, USA; 2 Children’s Hospital of Wisconsin, Department of Nursing Research, Milwaukee, WI 53226, USA; 3 Medical College of Wisconsin-Clinical and Translational Science Institute, Milwaukee, WI 53226, USA; amoosreiner@mcw.edu; 4 Epidemiology Program, University of Hawaii Cancer Center, Honolulu, HI 96789, USA; cjboushey@cc.hawaii.edu; 5 School of Electrical and Computer Engineering, Purdue University, West Lafayette, IN 47907, USA; ace@ecn.purdue.edu (E.J.D.); zhu0@ecn.purdue.edu (F.Z.)

**Keywords:** dietary assessment, mobile food record, 24-h recall, developmental disabilities, children, spina bifida, down syndrome, technology, pediatrics

## Abstract

Obesity prevalence is higher in children with developmental disabilities as compared to their typically developing peers. Research on dietary intake assessment methods in this vulnerable population is lacking. The objectives of this study were to assess the feasibility, acceptability, and compare the nutrient intakes of two technology-based dietary assessment methods in children with-and-without developmental disabilities. This cross-sectional feasibility study was an added aim to a larger pilot study. Children (*n* = 12; 8–18 years) diagnosed with spina bifida, Down syndrome, or without disability were recruited from the larger study sample, stratified by diagnosis. Participants were asked to complete six days of a mobile food record (mFR™), a 24-h dietary recall via FaceTime^®^ (24 HR-FT), and a post-study survey. Analysis included descriptive statistics for survey results and a paired samples *t*-test for nutrient intakes. All participants successfully completed six days of dietary assessment using both methods and acceptability was high. Energy (kcal) and protein (g) intake was significantly higher for the mFR™ as compared to the 24 HR-FT (*p* = 0.041; *p* = 0.014, respectively). Each method had strengths and weaknesses. The two technology-based dietary assessment tools were well accepted and when combined could increase accuracy of self-reported dietary assessment in children with-and-without disability.

## 1. Introduction

Assessment of an individual’s dietary intake is an essential component of the prevention and treatment of an abnormal weight status [1]. Details of dietary intake provide valuable information on an individual’s nutritional balance and dietary habits [2,3]. The interest in dietary assessment has heightened as the prevalence of obesity has increased. However, there is a lack of testing and development of tools focusing on children with developmental disabilities [4]. This is a critical oversight as the prevalence of obesity is often higher in children with developmental disabilities as compared to children who are typically developing [5].

Recommended assessment methods for dietary intake in children vary based on the child’s age and who is reporting [2,4]. Conclusions from a systematic review identified that the 24-h dietary recall reported by the parent for 4 to 11 year olds and dietary history reported by adolescents 16–21 years of age had the highest level of accuracy when compared to doubly labeled water [2]. Challenges to obtaining an accurate dietary assessment include social bias, the burden of time to complete, and the inability of the reporter to estimate portion sizes, identify food preparation methods, and recall foods consumed [1,6]. Currently there is no recommendation for dietary assessment in children with developmental disabilities.

Incorporating technology is thought to improve dietary intake accuracy, appeal to a younger generation, and reduce the burden placed on the reporter [7,8]. One option is the Technology Assisted Dietary Assessment™ (TADA™) system, an image-based dietary assessment system which uses the mobile Food Record™ (mFR™) app to collect images of eating occasions [9,10,11,12]. The app can be downloaded onto smart devices (e.g., mobile phone or iPad). The app allows individuals to record images before and after eating occasions and the images upload in real time to a cloud-based server along with contextual information, e.g., time.

The collection of dietary intake in real time is thought to reduce recall bias, provide additional information related to the individual’s eating behaviors, and increase convenience for the reporter [11]. Among 41 adolescents (11–15 years of age), use of the mFR™ was accepted by the majority [10]. Bathgate et al. [13] examined the feasibility of using the mFR™ in 59 adolescents and young adults (12–30 years of age; M = 21.5 (SD 4.6)) with Down syndrome. In this sample, 86% of the participants successfully recorded dietary intake using the mFR™ for a minimum of two days [13].

The objectives of this study were to assess the feasibility and acceptability of the mFR™ and a 24-h dietary recall conducted via FaceTime (24 HR-FT) among children with-and-without developmental disabilities. FaceTime is an app available on Apple^®^ products that allows individuals to use WiFi or cellular data to perform a call with video and audio capability. The estimates for total energy and macronutrient intakes were hypothesized to be similar between the methods. Results from this exploratory study can inform future studies to better assess dietary intakes among a vulnerable and understudied population.

## 2. Materials and Methods

### 2.1. Study Design and Sample

This study was part of a larger cross-sectional study examining energy expenditure assessment in 36 children with-and-without developmental disabilities [14]. This feasibility study was conducted as an added aim to the original study through an additional funding mechanism. Institutional Review Board approval was granted through a Midwestern Children’s Hospital and parents and children provided written informed consent and assent. Study visits were conducted within a Translational Research Unit funded by the Clinical and Translational Science Institute of Southeast Wisconsin.

Participants included 12 of the original 36 children diagnosed with Down syndrome, spina bifida, or no developmental disability. A sample of 12 participants was determined based on funding and feasibility design. Based on a completed permission to contact form from the parent study, participants stratified by diagnosis were randomly recruited for this study.

### 2.2. Measures

#### 2.2.1. Dietary Assessment (mFR™)

**Figure 1:**
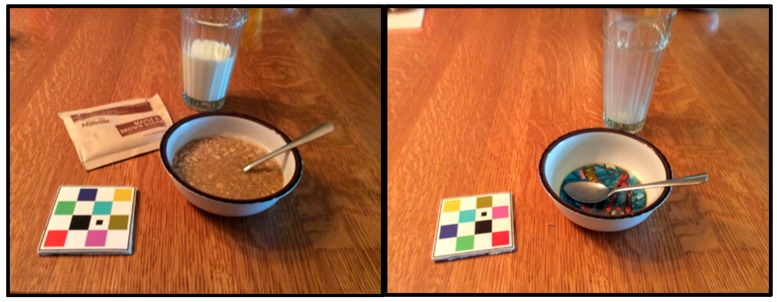
Before and after Mobile Food Record**™** images with the fiducial marker.

Study participants were provided a mini iPad^®^ (iOS version 9.3, Apple Inc., Cupertino, CA, USA) with the mFR™ and FaceTime app. These community dwelling children were asked to obtain images before and after all meals/snacks for a 24-h period for a total of six days (4 weekdays and 2 weekend days) of their choice over a two-week period. Data collection occurred during late summer and fall seasons. Participants were instructed to eat as usual. The child and parent were provided training and practiced using the mFR™ with a cafeteria meal. Training focused on technical issues, such as the need to incorporate the provided checkered fiducial marker in the eating scene to aid volume estimation (Figure 1), and problem solving for common mealtime issues such as having seconds or placement of food labels within the eating scene to assist the intake analysis. Parental assistance was recommended to be used as needed. Pre- and post-eating occasion images were automatically uploaded to a secure cloud-based server. A trained team member used the images to enter the food intake and amounts using Nutrition Data Systems for Research, a computer-based software application [15].

#### 2.2.2. Dietary Assessment (24 HR-FT)

Participants were instructed that each subsequent day following the mFR™, they would be asked to complete a 24-h dietary recall conducted via the FaceTime app on the provided mini iPad. Scheduling of the FaceTime calls were predetermined with the family. The 24 HR-FT was conducted by a dietitian trained to use a multiple-pass method which included extracting forgotten foods and detailed portion sizes. During training, participants were provided with a set of standard measuring cups and spoons, a deck of cards, and 2-dimensional portion size tools for use as a reference during the recalls. Parental assistance was recommended to be used as needed. At the time of the 24 HR-FT, the interviewer did not access or preview the mFR™ images.

Following the six days of dietary intake recording by the mFR™ and the 24 HR-FT, the child and parent were asked to complete a post-study survey. The survey included questions on use of parental assistance and details specific to each method.

### 2.3. Statistical Analysis

The dietary intake data collected using the mFR™ and 24 HR-FT were entered and analyzed using the Nutrition Data System for Research software version 2015 developed by the Nutrition Coordinating Center (NCC), University of Minnesota, Minneapolis, MN [15]. The survey responses were analyzed using descriptive statistics. Daily intake of energy (kcal), carbohydrates, fats, and proteins were compared between the methods with a paired samples *t*-test. Statistical analyses were performed using SPSS (IBM SPSS Statistics Version 25; Chicago, IL, USA). Statistical significance was set at a *p*-value < 0.05.

## 3. Results

### 3.1. Sample Characteristics

The cohort (*n* = 12) equally represented the three groups (spina bifida (*n* = 4), Down syndrome (*n* = 4), and no disability (*n* = 4)) with ages between 8 and 18 years old (M = 13.17; SD 3.35) and included six boys and six girls.

### 3.2. Feasibility and Acceptability

The six days of recording dietary intake with the mFR™ and 24 HR-FT were successfully completed by 12 of the 12 study participants. All 12 children were willing to use the mFR™ and participate in multiple 24 HR-FT in a future study. See Table 1 for additional results. All parents who completed the survey (*n* = 11) were women. Six parents reported assisting their child with the mFR™ and eight assisted with the 24 HR-FT.

### 3.3. Energy and Dietary Macronutrients

Significant differences were identified for kcals per day from 24 HR-FT (M = 2020, SD = 626) as compared to mFR™ (M = 1855, SD = 508), *t* (11) = 2.32, *p* = 0.041 and for protein (g/day) from 24 HR-FT (M = 80, SD = 27) as compared to mFR™ (M = 69, SD = 19), *t* (11) = 2.92, *p* = 0.014 with the 24 HR-FT assessment being higher for both. No significant differences were reported for dietary fats (g/day) between the 24 HR-FT (M = 81, SD = 32) and mFR^TM^ (M = 75, SD = 25), *t* (11) = 1.29, *p* = 0.223. Similarly, no significant differences were identified for dietary carbohydrates (g/day) when comparing the 24 HR-FT (M = 250, SD = 73) and mFR^TM^ (M = 233, SD = 70), *t* = 2.0, *p* = 0.071.

### 3.4. Post Hoc Observations

Strengths of the mFR™ included the ability to capture intake not identified by the 24 HR-FT, which was commonly either a snack or non-nutritive item. Weaknesses included the limited ability to extract details from the images, e.g., preparation and food density. Strengths of the 24 HR-FT included the ability to probe and expand on questions related to types of foods and meal components. Weaknesses of the 24 HR-FT included the child’s inability to accurately remember intake, identify food preparation details, and estimate portion sizes. Parental involvement was highest among children with Down syndrome and all groups in the age range of 8 to 12 years. Of the parents who assisted their children, there was a generalized reduced awareness of complete dietary intake for the child.

## 4. Discussion

In this feasibility study, the mFR™ and 24 HR-FT dietary assessment methods were both well accepted by children with and without developmental disabilities. Requesting the use of both methods for a total of six days within a two-week timeframe was feasible for both child and parent schedules. This expanded on what was reported by Bathgate and colleagues [13] who tested the feasibility of using the TADA mFR™ in a slightly older sample of individuals with Down syndrome. In their study, 86% (51/59) of the sample successfully recorded nutritional intake with the mFR™ for a minimum of two days [13], whereas 100% of the sample in the current study successfully collected both the image recordings for the mFR™ and the 24 HR-FT for a total of six days. Notable differences between these studies were that the sample in the current study was smaller, younger, and included children with spina bifida and without developmental disabilities. In addition, the current study was able to provide information on parental assistance with the assessment tools. Benefits and limitations of each of the dietary assessment methods became evident following execution of this study protocol.

A notable strength of the mFR™ was the ability to capture snacks or non-nutritive food choices that were often not reported in the 24 HR-FT. This finding was similar across all participant groups and ages. The omission of this intake in the recalls may have been due to issues of memory, mindless eating, or social desirability bias.

Challenges related to the mFR™ included difficulty in identifying food items from the uploaded images on the web server. Having a single 2-dimensional image did not consistently provide sufficient details regarding the food item, portion size, or the preparation methods. A dietitian completing a brief review with study participants regarding items needing additional information as done by Kerr [11] and Bathgate [13] could address these issues. The mFR™ used a fiducial marker to assist the human eye to estimate volume but potential for error was still present. These challenges are not specific to the mFR™. Food supplies and systems have produced an infinite number of possible nutrient compositions per food item creating challenges for any assessment method.

Completion of the 24 HR-FT was well accepted by the study participants. The use of FaceTime to complete the recall proved to be convenient and offered the investigator and reporter face-to-face interview benefits. Recall appointments were able to take place anywhere there was an internet connection decreasing the burden to participants. The face-to-face interview potentially reduced misreporting by allowing the investigator to observe social cues including eye movements and facial expressions, which assisted in the determination of when to probe for further information.

A common limitation when using the recall method is the inability for the reporter to remember all food consumed. When recalling independently, participants sometimes did not remember intake that they had documented with an image the day before. These image confirmed differences might have contributed to the larger amount of inaccuracies in the children between 8 and 15 years of age. In addition to difficulties recalling consumed items, all participants struggled to describe how food items were prepared, provide food details (e.g., low-fat), and estimate portion sizes. However, the option of using the provided measuring cups and spoons lessened this problem. When given the option of having parental assistance with recalls, children with Down syndrome and all children between 8 and 12 years of age employed this. This may be related to Down syndrome having a higher potential of cognitive impairment and a poor working memory or it may be indicative of this age group. When used, parental assistance was not always useful. Parents were often unaware of specifics related to what their child ate throughout the day. This is not unexpected as food is often consumed outside of the home or can be eaten independently within the home.

When comparing energy and macronutrient intakes between the two methods, dietary fats and carbohydrates were consistent with each other, but energy (kcals) and protein intake were significantly different between the methods with the 24 HR-FT measuring higher for both. The rationale for this difference is uncertain but may stem from the challenges related to extracting details from the TADA™ images or the added benefit of being face-to-face for the 24 HR-FT. As noted above a review process after collecting the images might address this [11,13]. Further study would be needed with larger sample sizes to confirm if these remain consistent findings.

The intent of the study was to compare two novel methods of dietary assessment in children with and without developmental disabilities. Having the ability to perform each method back-to-back not only allowed the authors to compare the methods but it also highlighted how unique attributes of each method could be synergistic if used together. During analysis, it became evident that the TADA™ images captured intake that was not identified by the child during the 24 HR-FT, which may alleviate issues related to the inability to recall food consumed the previous day. In addition, the 24 HR-FT could provide the trained interviewer the opportunity to ask questions or to use props to gain valuable details related to the food in the TADA™ images. While our team did not preview the TADA™ images prior to the subsequent 24 HR-FT, deliberately replicating the sequence of these two methods and using the 24 HR-FT to complement the mFR™ could be extremely valuable and is recommended for future studies.

Study strengths were the inclusion of children with disabilities, the use of the same food composition table, and that a single team member entered all data for analysis. Particular limitations include the small sample size and cross-sectional design that limits the generalizability of study findings. Also, the errors inherent with interpreting dietary information for data entry to a food composition table and lack of an objective biomarker.

## 5. Conclusions

This feasibility study provided valuable information in a vulnerable subset of children who have a higher prevalence of obesity and could be applied to all children regardless of disabilities. The mFR™ and conducting multiple pass 24-h dietary recalls over FaceTime are two novel methods of assessing dietary intake. The use of technology appeared to benefit acceptance and willingness to complete the tools in a sample of children with-and-without developmental disabilities and their parents. Each tool had its own strengths and weaknesses that could leverage the other. The combination of methods may increase the accuracy of self-reported dietary assessment in children and is recommended for further study in larger samples.

## Figures and Tables

**Table 1:** Child post-study survey result.

**Mobile Food Record (mFR™)**	
Willing to use TADA mFR™ in future	12/12 (100%)
Ease of use (1-very easy; 10-very difficult)	1 (75%); 3 (8.3%); 4 (8.3%); 9 (8.3%)
Screen easy to read	10 (83.3%) strongly agree and 2 (16.7%) agree
Easy to enter information	8 (66.7%) strongly agree and 4 (33.3%) agree
Information provided was accurate	10 (83.3%) strongly agree and 2 (16.7%) agree
Interfered with daily activities	4 (33.3%) strongly disagree; 7 (33.3%) disagree and 1 (8.3%) agree
**24-h Recall by FaceTime (24 HR-FT)**	
Willing to use 24 HR-FT in future	12/12 (100%)
Ease of use (1-very easy; 10 very difficult)	1 (83.3%); 2 (8.3%); 4 (8.3%)
Easy to recall food	4 (33.3%) strongly agree and 8 (66.7%) agree
Information provided was accurate	7 (58.3%) strongly agree and 5 (41.7%) agree
Interfered with daily activities	5 (41.7%) strongly disagree; 6 (50%) disagree and 1 (8.3%) agree

TADA™: Technology Assisted Dietary Assessment.
